# Expansion of Impervious Surfaces and Their Driving Forces in Highly Urbanized Cities in Kyrgyzstan

**DOI:** 10.3390/ijerph17010362

**Published:** 2020-01-05

**Authors:** Gulkaiyr Omurakunova, Anming Bao, Wenqiang Xu, Eldiiar Duulatov, Liangliang Jiang, Peng Cai, Farkhod Abdullaev, Vincent Nzabarinda, Khaydar Durdiev, Makhabat Baiseitova

**Affiliations:** 1 State Key Laboratory of Desert and Oasis Ecology, Xinjiang Institute of Ecology and Geography, Chinese Academy of Sciences, Urumqi 830011, China; omurakunovagulkaiyr@gmail.com (G.O.); xuwq@ms.xjb.ac.cn (W.X.); liangliang.jiang@ugent.be (L.J.); peng.cai@ugent.be (P.C.); vincentnzabarinda@yahoo.com (V.N.); durdiyev87@inbox.ru (K.D.); 2 Key Laboratory of GIS & RS Application Xinjiang Uygur Autonomous Region, Urumqi 830011, China; 3 University of Chinese Academy of Sciences, Beijing 100049, China; abdullaevfarkhod87@gmail.com; 4 Kyrgyz National University Named after Jusup Balasagyn, 547 Frunze, Bishkek 720033, Kyrgyzstan; mbaiseitova@inbox.ru; 5 Institute of Geology, National Academy of Sciences of the Kyrgyz Republic, 30 Erkindik, Bishkek 720040, Kyrgyzstan; e.duulatov89@gmail.com; 6 Department of Geography, Ghent University, 9000 Ghent, Belgium; 7 Key Laboratory of Water Cycle and Utilization in Arid Zone, Urumqi 830011, China; 8 Ministry of Water Resources of the Republic of Uzbekistan, Scientific Research Institute of Irrigation and Water Problems, Tashkent 100187, Uzbekistan

**Keywords:** impervious surfaces, urban, driving factors, supervised classification, landscape expansion index

## Abstract

The expansion of urban areas due to population increase and economic expansion creates demand and depletes natural resources, thereby causing land use changes in the main cities. This study focuses on land cover datasets to characterize impervious surface (urban area) expansion in select cities from 1993 to 2017, using supervised classification maximum likelihood techniques and by quantifying impervious surfaces. The results indicate an increasing trend in the impervious surface area by 35% in Bishkek, 75% in Osh, and 15% in Jalal-Abad. The overall accuracy (OA) for the image classification of two different datasets for the three cities was between 82% and 93%, and the kappa coefficients (KCs) were approximately 77% and 91%. The Landsat images with other supplementary data showed positive urban growth in all of the cities. The GDP, industrial growth, and urban population growth were driving factors of impervious surface sprawl in these cities from 1993 to 2017.Landscape Expansion Index (LEI) results also provided good evidence for the change of impervious surfaces during the study period. The results emphasize the idea of applying future planning and sustainable urban development procedures for sustainable use of natural resources and their management, which will increase life quality in urban areas and environments.

## 1. Introduction

Controlled and planned urban expansion is a surety of a healthy human society. However, unplanned urban expansion is a human-made disaster. Researchers have noted dramatic urban expansion [1] from previous studies, indicating that approximately 5% [2] of global land was converted into the impervious surface area. According to the United Nations, more than half of the world’s population inhabit urban areas, and this number will be expected to reach more than 68% by 2050 [3,4,5,6]. Anthropogenic activities strongly influence ecosystems in urban areas, with more attention currently being paid to monitoring land use and land cover changes [7]. It is more important to study the spatial patterns and their characteristics relating to land use and land cover (LULC) to understand the effects of human activities on ecology conditions and urban environments [8,9]. Change in urbanization has shown a clear impact on people and their environments [3,4]. It can be argued that expansion (urbanization) offers many benefits to human society, such as stimulating the economy, developing societies, and creating educational and cultural centers that increase the economic productivity of any country [10,11]. However, urbanization also leads to significant changes in landscapes and ecosystems, and poses many problems, including environmental degradation, which creates risks for environmental and socio-economic activities [8,10]. The close internal relationship between GDP and urban expansion has revealed that the current Kyrgyzstan economy is highly dependent on existing natural resources, especially on land. This dependency requires an urgent exploration of the applied structure to decouple the connection between economic development and urban growth. It is important to alter the economic development from a wide design perspective, depending on large amounts of resources [12]. Urban land cover information provides useful evidence regarding ecological, environmental, and climatic effects on different spatio-temporal scales [13,14]. Therefore, accurately describing the consistent pattern of impervious surface expansion is a prerequisite for the collective development of urban and regional sustainability plans.

After the dissolution of the Soviet Union (USSR), Kyrgyzstan experienced rapid urban expansion, especially in the capital, Bishkek. Previous studies have demonstrated that Kyrgyzstan is facing country-wide urbanization, significant urban accumulation, and the development of zones with a long human history and a comparatively high level of economic prosperity in Bishkek. Predictably, urban expansion patterns were exposed by examining the driving forces [15].

Similar to other countries of the world such as China and Russia, urbanization is becoming a severe challenge for Central Asia, which is experiencing dynamic economic and demographic growth. After independence, the Central Asian countries developed their own political structures and economic reforms in many different ways [16], which has had a significant impact on the development of urban processes. The Soviet period in the history of the Central Asian countries was characterized by a significant increase in urban populations, as well as in overall population. The establishment of new cities and their expansion, social infrastructure, and development of agricultural and mining industries have contributed to the economies of the region [17]. The processes involved in the urbanization of Kyrgyzstan depend on the socio-economic situation of the country; a recession or a slowdown in economic growth causes a decrease in the rate of urbanization, and vice versa. In turn, industrialization followed railways, which were laid to ensure access to mineral resources [18]. From 1941 to 1942, approximately 30 factories and plants were moved to Kyrgyzstan and were partially reoriented to military purposes. They were located mainly in the Chui and Bishkek regions [17]. In 1990, almost 38% of the population of Kyrgyzstan migrated into cities due to the changing behavior of traditional economic activities and lifestyles. Kyrgyzstan experienced a significant population outflow after 1991 [19].

Large cities, such as Bishkek, Osh, and Jalal-Abad, and to a lesser extent regional centers, face a high level of internal migration [20]. This phenomenon directly affects the economic, social, and cultural aspects of the population. Small cities and settlements in the country with an outflow of young people also experience significant difficulties in socio-economic development. Therefore, the government of Kyrgyzstan, in its new program “Cities—points of economic growth”, drew attention to these problems and intends to use these resources on the ground, creating jobs and spaces [21]. The driving forces of unplanned impervious surface expansion are divided into two categories: potential and direct factors. Direct factors include development in terms of infrastructure, settlements, and industrial development, as mentioned by Lu et al. and Huang et al. [22,23]; and potential factors include technological, economic, population, policy, and natural factors [24,25,26]. Due to constraints in the availability of continuous statistical data, quantitative analyses are frequently used for impervious surface analyses [27]. However, quantitative spatio-temporal analysis is insufficient for strategic scientific proposals and their planning.

Impervious surfaces are the key indicator for determining the urbanization growth rate in terms of the quality of urban development [23]. Impervious surfaces represent materials that do not absorb water or moisture, including roads [28], parking lots, and most urban infrastructures, highways, and sidewalks, which resist rainwater seepage to recharge groundwater aquifers [27]. Instead, rainwater flows quickly into nearby streams, causing unnaturally large and sudden flows that contribute to stream erosion [29].

Several studies have confirmed the application of Geographic Information System (GIS) and remote sensing (GIS/RS) in the context of urban planning and development. GIS/RS studies have been presented to date, and are the main analysis technique used to asses urban changes, as well as for growth modeling [30,31,32], LULC assessment [33,34], and urban heat island investigations [35,36,37]. Specifically, RS-based multitemporal land use change data offer evidence that can be used to measure the physical changes of the LULC area. Additionally, detailed and accurate land use statistics provides useful information for sustainable and future urban planning [38]. Therefore, it is important to calculate the rate of change in patterns and LULC types to predict upcoming changes in urban areas and their development [33]. The maximum likelihood classifier fits a normal distribution to each class of radiometric values. It classifies each class (infrastructure classes can be verified visually) correctly as compared to other classification techniques, such as random forests or minimum distances [39]. Moreover, the maximum likelihood classifier provides the best results of urban expansion based on normal distribution. Therefore, this study used a maximum likelihood classification technique for urban expansion and impervious surface analysis. Geographic information system (GIS) and remote sensing (RS) data and tools are frequently applied in the field of urban expansion studies, mainly focusing on urban area extraction and spatio-temporal land cover classification to measure expansion dynamics [40,41]. Different techniques, such as change analysis (land use) and spatial modeling, were applied in this study. These methods regularly require the quantitative and qualitative application of GIS and RS data to extract desired results from spatial and temporal datasets [42]. The specific objectives of the study are given below:

- To analyze the spatial and temporal characteristics of impervious surface expansion from 1993 to 2017.
- To examine and calculate land use and land cover change in the study period, along with developed land use and land cover maps.
- To analyze and identify urban growth types using the Landscape Expansion Index (LEI) index.
- To analyze the driving factors of impervious surfaces.

The majority of previous studies used simple image classification methods on a small scale, or only focused on satellite data. Therefore, in order to fill this knowledge gap, this study is mainly focused on satellite data, using Landsat images as input data to measure and analyze the expansion patterns of impervious surfaces from 1993 to 2017 (1993–2000, 2000–2010, and 2010–2017) in three cities of Kyrgyzstan via a supervised image classification technique and the landscape expansion index (LEI)) with different driving forces (GPD, industrial growth, and population) of urban expansion, which directly impact the livelihoods of the population in the study area.

## 2. The Study Area

**Figure 1:**
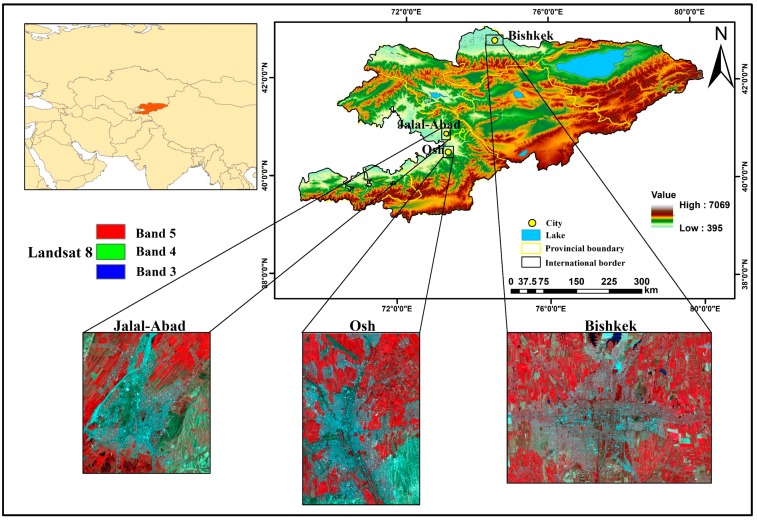
Location of the study area.

The cities of Bishkek, Osh, and Jalal-Abad in Kyrgyzstan were selected as the study area; the geographical locations are (42°52′29.02″ N, 74°34′14.30″ E), (40°30′50.39″ N, 72°48′57.95″ E), and (40°55′57.34″ N, 72°58′53.19″ E), respectively (Figure 1).

In Kyrgyzstan, 33.9% of the total population live in urban areas and 66.1% live in rural areas. The population density averages 31 people per square kilometer. Bishkek, Osh, and Jalal-Abad are the main cities of Kyrgyzstan, having an area of approximately 678.6 km^2^, 193.4 km^2^, and 116 km^2^, respectively [43]. These three cities are among the top three cities in the country regarding population and development. These cities are experiencing urbanization along with pressure from local migration, as people are looking for a better lifestyle and basic life necessities, such as education, health, and employment. This was the main reason to select these three cities as a case study for urban expansion and impervious surface analysis. Bishkek is the capital and largest city, and is important as the center of administration, economy, culture, and transportation [43]. Kyrgyzstan consists of seven provinces: Batken, Jalal-Abad, Issyk-Kul, Naryn, Osh, Talas, and Chui; the capital is Bishkek. Kyrgyzstan measures approximately 925 km from west to east and approximately 454 km north to south; it borders China, Tajikistan, Uzbekistan, and Kazakhstan, and has an approximate land cover area of 199,900 km^2^ [44]. Three cities with different urban settings located in different geographical locations and climate regions are used as our study area. Bishkek is in the north of Kyrgyzstan, in the Chui Valley at the foothills of the Tian Shan, 40 km north of the Kyrgyz ridge at an elevation of 700–900 m above sea level and 25 km from the border with Kazakhstan. It is a particular administrative unit and is a city of republican subordination; its former names are Pishpek (until 1926) and Frunze (1926–1991). The climate of Bishkek takes an extreme southern position in the continental climate of temperate latitudes. The climate is sharply continental in Bishkek; the average annual air temperature is +11.3 °C. The coldest month is January (−10 °C), and the warmest month is July (+38 °C). The average monthly relative humidity levels are 60%, 44%, and 74% in January, June, and July, respectively. Ala-Archa and Alamedin flow down from the southern mountains and the east–west flows of the Big Chui canal flow north of Bishkek [44]. The administrative boundary of Bishkek city is mainly divided into four districts, namely Oktyabr, Pervomai, Sverdlov, and Lenin. This region is the central hub for political, economic, tourism, and scientific research, and is a cultural center and transportation hub. It is located in the north of the country, in the Chui region. Bishkek today is the largest and most developed city in the state [45].

Osh is a city of republican subordination in Kyrgyzstan, and it is the administrative center of the Osh region. It is located in the southern region of the country and 300 km away from Bishkek in the southwest direction. The city is located in the eastern part of the Fergana Valley at the outlet of the Ak-Buura River (Akbuura) and the foothills of the Alai Range at an altitude of 870–1110 m. The second most populous city in the country, it is also called the “southern capital”.

Jalal-Abad, the third-largest city in Kyrgyzstan, is the administrative center of the Jalal-Abad region. Jalal-Abad is located in the foothills of the Tian Shan at the base of the small Ayub-Too Mountains at an elevation of 763 m above sea level in the Kogarth Valley. The distance to the capital of Kyrgyzstan (Bishkek) is 560 km. Jalal-Abad is located 100 km from the southwest border of the country [46].

## 3. Materials and Methods

### 3.1. Dataset and Preprocessing

In this study, all the raw images of Bishkek, Osh, and Jalal-Abad were acquired from the United States Geological Survey (USGS) data archive (https://earthexplorer.usgs.gov/); data from 1993, 2000, 2010, and 2017 with less than 10% cloud cover for the selected area (24 years) were used in this study. Datasets from different scanners (Landsat 5 Thematic Mapper (TM), Landsat 7 Enhanced Thematic Mapper Plus (ETM+), and Landsat 8 Operational Land Imager (OLI)–Thermal Infrared Sensor (TIRS)) were downloaded from the web portal [47]. Each satellite tile of Landsat data was reprojected from World Geodetic System 1984 (WGS 84) to the Universal Transverse Mercator (UTM) Zone 43N system with the project transformation tool [24]. More details about the data are given in Table 1. The population, industrial, and gross domestic product (GDP) data were collected from statistical yearbooks from the National Statistical Committee of the Kyrgyz Republic [48].

To extract better results from the data, image preprocessing techniques, such as radiometric calibration and atmospheric correction, were applied to the downloaded images in Environment for Visualizing Images (ENVI) 5.1 before applying the classification algorithms [26]. Generally, Landsat satellite images did not require geometric correction [49]. The remaining part of the raw data came from preprocessing Landsat images, including atmospheric and radiometric calibration performed for further processes. However, we used only multispectral bands, including Blue (B), Green (G), Red (R), Near Infrared (NIR), Short Wave InfraRed 1 (SWIR1), and Short Wave InfraRed 2 (SWIR2). Landsat images, atmospherically and radiometrically calibrated using fast line-of-sight atmospheric analysis of spectral hypercubes (FLAASH) atmospheric correction model, were applied using the Environment for Visualizing Images (ENVI) software version 5.1 [49,50,51]

For data validation and verification, Sentinel-2 data were gathered from the USGS web portal (glovis.usgs.gov) for the year 2017 [52]. There are 13 spatial bands of Sentinel-2 data, in which four spectral bands have a 10 m spatial resolution, six bands have 20 m of spatial resolution, and the remaining 3 have 60 m [53]. For validation and verification of Landsat data, Sentinel-2 bands were stacked, such as B02, B03, B04, and B08. The scope of Sentinel-2 imagery was used for image classification of forestry, agriculture, land resource management, and environmental monitoring.

### 3.2. Method

This study used different types of software tools for image analysis and processing to obtain the desired results from satellite images, namely ArcMap 10.2 (https://www.esri.com) and ENVI 5.1 (https://www.harrisgeospatial.com).

**Figure 2:**
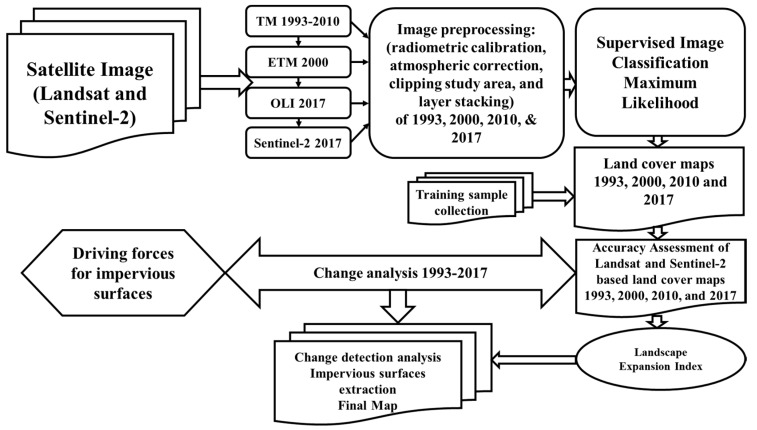
Flowchart of urban expansion method.

ArcMap 10.2 was used to process 30 m spatial resolution Landsat images and make a study area map, as well as to for supervised classification to extract impervious surface area maps of four datasets, namely for the years 1993, 2000, 2010, and 2017. Furthermore, the final maps were computed with reclassified maps (i.e., impervious and non-impervious surfaces, land use, and the resultant maps were obtained for the landscape expansion index by analyzing urban growth types for 1993–2000, 2000–2010, and 2010–2017 maps) Figure 2.

For the impervious surface mapping, the most advanced and recent classification method was used—supervised maximum likelihood classification. This classification procedure includes spectral values, as well as image interpretation elements (such as shape, size, and texture) and compression of image elements. Multitemporal land cover maps for the study area were developed with the help of current and temporal resolution datasets. Different types of algorithms with overlay techniques were applied to computed change pockets within different urban sections. Based on visual interpretation and reflectance values, the Geospatial tool (used for spatial analysis, segmentation, and classification) in ArcMap 10.2 was used for mapping and impervious area analysis.

The required training samples generally included four broad categories for supervised classification: built-up areas (impervious surface areas), natural vegetation, bare land, and water, which were extracted for the specific period. Among them, urban land included high-reflectivity buildings (cement or concrete roofs) and low-reflectivity buildings (mainly brick and tile roofs in the old town), individual buildings and groups (clusters) of building, roads, airports, and others, which were encompassed in the impervious surface area [42]. Natural vegetation (green spaces) covered most of the growing season in summer, so all natural vegetation was classified into one category. Bare land mainly refers to the land without vegetation throughout the year, including deserts, wasteland, and fallow land, which were classified into one category, along with water bodies being classified into one class [54]. The extensively used maximum likelihood technique was used to differentiate different types of land cover [34]. Supervised classification techniques are regularly used for quantitative analyses [24]. The maximum likelihood classifier considers that radiometric values in each class fit a normal distribution. The maximum likelihood estimation correctly classified more of the infrastructure classes than the other two techniques, and this could be verified visually. Random forests or minimum distances did not achieve this aspect of the evaluation due to the lack of support vectors [39]. Additionally, the best result was provided by the maximum likelihood classifier for urban expansion on the basis of normal distribution. In this classification, we took a number of spectral signatures for the specified locations (training samples) from the image to classify all pixels into a number of classes [42]. The method used in the study was based on the integration of the impervious surface area, GDP, and industrial and population data.

### 3.3. Accuracy Assessment and Data Validation

In an analysis, accuracy assessment (AA) is an important step in classification. For every classification test, 30 samples were used. In total, 120 random points were separated from each class for AA from 1993, 2000, 2010, and 2017 (used to determine the similarity of two images), and from Sentinel-2 satellite images for 2017 [25,55,56]. The mutual information from the error matrix was used to assess the accuracy of the supervised image classification [57]. Some critical measurements were computed using the error matrix method [58]; that is, the producer’s accuracy (PA), overall accuracy (OA), and user’s accuracy (UA). Qualitative performance appraisals were adopted on the basis of this technique. The PA, UA, OA, and kappa coefficient (KC) values were calculated with a confusion matrix from the detected results, and the ground truth data were collected from different locations in the study area [22,59]. The PA is the error emission in the classified images, and the UA is the reliability; the probability of a pixel on the map represents that of the same category on the ground. The OA is the total number of pixels in the error matrix divided by the number of correctly classified pixels (on the diagonal of the error matrix). The KC is calculated by measuring the agreement between the reference data and the classified map (Equation (1)).

(1) $$K\;=\;\frac{N\sum_{i\;=\;1}^{r}x_{ii}\;-\;\sum_{i\;=\;1}^{r}\left(x_{i+})(Xx_{+i}\right)}{N^{2}-\sum_{i\;=\;1}^{r}\left(x_{i+})(Xx_{+i}\right)}$$

where *K* is the KC value, *N* is the total number of observations, *X_ii_* is the observation in row *i* and column *i*, *X_i_* is the marginal total of row *i*, and *X + i* is the marginal total of column *i*.

AA is a necessary component of any classification task. It compares the classified image to another information source, namely the ground-truth information. The commonly used method for this is to survey a detailed classified map and collect a set of random ground points to compare with the detailed classified map using a confusion matrix. However, this method is a two-step process to compare the study results gathered from different classification techniques with those from the training samples. There may not be enough ground truth data in the same image to allow the classification. These workflows were implemented with three geoprocessing tools [60].

The results of the AA for the classification of 1993, 2000, 2010, and 2017 satellite images were assessed through different AA procedures (Equation (1)). A number of trial samples from the data used for the classification were validated with Sentinel-2 data for 2017. The indices computed for the evaluation of all classifications were the OA, KC, PA, and UA. The AA results are shown in Table 2.

### 3.4. Landscape Expansion Index

**Figure 3:**
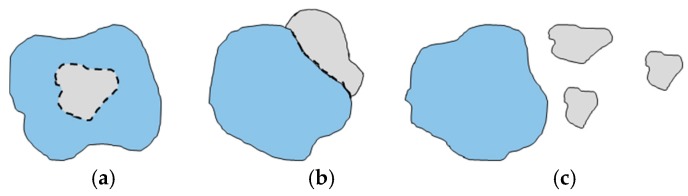
Types of urban growth: (**a**) infilling growth; (**b**) edge expansion; (**c**) outlying growth.

There are apparent limitations to traditional landscape indicators for analyzing urban expansion in fast-growing regions. The most important limitation is that they can quantitatively reflect the patterns in the landscape and their distribution at only one point in time [61]. However, the landscape expansion index (LEI) can be used to define the process of landscape changes within more than two points [62]. According to previous studies, the LEI [61] identifies three growth types (Figure 3): (a) infilling growth, (b) edge expansion, and (c) outlying growth [63].

The LEI was calculated using analysis tools (Erase and Intersect data management tools) in ArcGIS as follows:

(2) $$LEI\;=\;\frac{\;Lc}{P}$$

where *Lc* is the length of the common boundary and *P* is the perimeter of the newly developed urban land patch. The LEI value indicates (a) infilling when LEI > 0.5, (b) edge expansion when LEI < 0.5, and (c) outlying expansion when LEI = 0. Infilling growth that occurs within urbanized, undeveloped pixels is converted to an urban area. Edge expansion refers to the newly developed urban area spreading out from the fringes of existing urban patches. Outlying growth refers to the change from nonurban to urban that occurs away from existing urban areas [62,64].

## 4. Results

### 4.1. Impervious Surface area for Different Years in Three Cities

**Figure 4:**
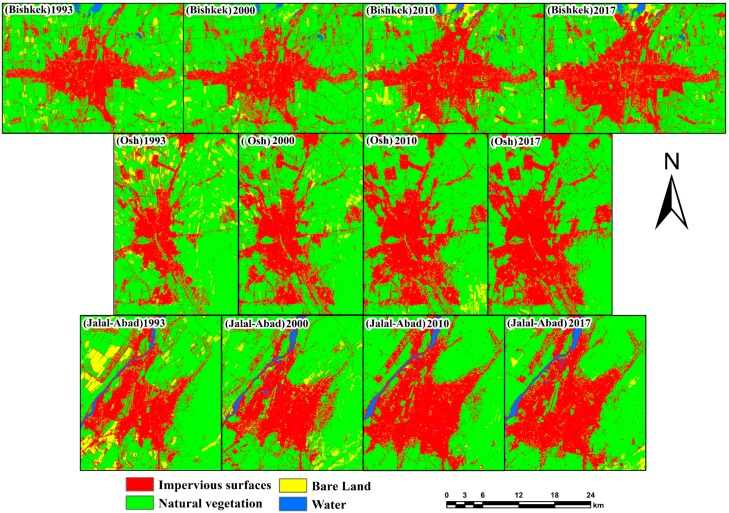
Impervious surface areas for different years.

**Figure 5:**
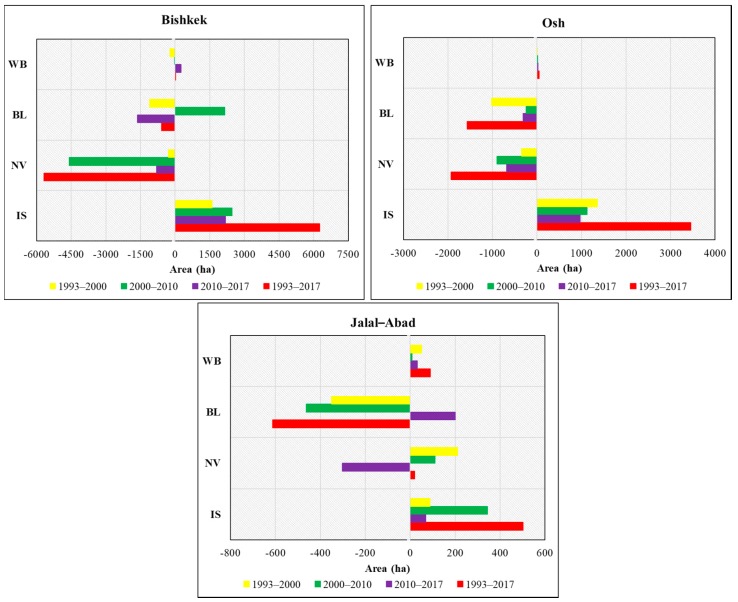
The bar charts showing the changes in the area covered during the study period. Note: IS—impervious surface; NV—natural vegetation; BL—bare land; WB—water body.

The land use and land cover classification of the study area was classified using Landsat images within the study periods of 1993, 2000, 2010, and 2017, and is clearly represented and mapped with statistics in Figure 4 and Figure 5.

Figure 4 and Figure 5 represent the four land classes in Bishkek, Osh, and Jalal-Abad for four different periods (1993–2000, 2000–2010, 2010–2017, and 1993–2017). From the study results, it is revealed that the impervious surface area had increased and the natural vegetation and bare land areas had declined significantly during the study period in Bishkek, followed by urban area increase. The remaining land use classes did not change significantly according to the bar chart (Figure 5), showing that impervious surfaces increased by 6255.54 ha, natural vegetation and bare land declined by 5686.11 ha and 591.75 ha, respectively, and water bodies increased by 23.13 ha in Bishkek from 1993 to 2017. In Osh from 1993 to 2017, impervious surfaces increased by 3452.67 ha, natural vegetation and bare land decreased by 1931.93 ha and 1566.54 ha, respectively, and water bodies increased by 45.81 ha. In Jalal-Abad from 1993 to 2017, impervious surfaces increased by 502.2 ha, natural vegetation increased by 19.62 ha, bare land decreased by 611.64 ha followed by urban area increase, and waterbodies increased by 89.82 ha.

The study results revealed that the overall KCs for 2017 are 78.9%, 77%, and 91% for Bishkek, Osh, and Jalal-Abad, respectively. For Bishkek (1993–2017), the OA values varied from 82.5% to 94.2%, with KCs of 77% to 92%; for Osh (1993–2017), the OA values varied from 82.5% to 96.7%, with KCs of 77 to 96%; and for Jalal-Abad (1993–2017), the OA values varied from 80% to 93.3%, with KCs of 73 to 91%. It seems from the results that the lowest PA and UA could not be achieved for the same (land) classes for a different set of cities. However, high PAs and UAs were frequent for water bodies because the study focused on impervious surface analysis. The OA and KCs for the Landsat 8 OLI and Sentinel-2 data validation period of 2017 for the three cities in the study area are also represented in Table 2.

The study results for the Sentinel-2 data revealed that the overall classification accuracies achieved were 94%, 88%, and 92%, and the overall KCs were 92%, 84%, and 89% for the 2017 image classifications in the three cities. The OAs achieved were 84.2%, 82.5%, and 93.3%, respectively, for Bishkek, Osh, and Jalal-Abad for 2017. Similarly, the overall KCs for 2017 were 78.9%, 77%, and 91% for Bishkek, Osh, and Jalal-Abad, respectively. The PAs and UAs for each class were as follows: As the results of the two datasets showed, we can say that Sentinel data are the most accurate (Table 2). In addition, for 2017, the choices attained for PA were 97%–100%, 67%–100%, and 73%–100%, where UAs were 93%–100%, 68%–100%, and 78%–100%, respectively, for the cities of Bishkek, Osh, and Jalal-Abad. The results show that the lowest PA could not be reached for the same land classes for different years with different cities.

### 4.2. Impervious and Nonimpervious Surface Map for the Three Main Cities

**Figure 6:**
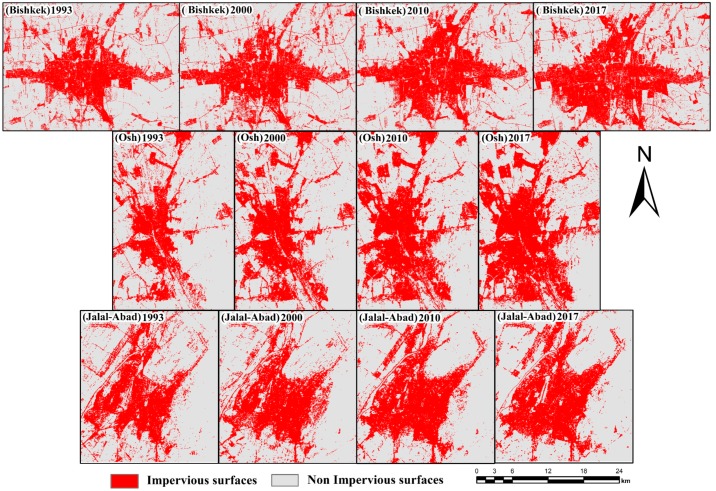
Map of impervious and nonimpervious surfaces for the three main cities.

This study is focused on change analysis of land use, and the land use and land cover maps were reclassified into two classes, namely impervious surfaces and nonimpervious surfaces, as shown in Figure 6. The classes were derived from the reclassification process for the 1993, 2000, 2010, and 2017 images for the study period. From the statistical analysis and visual measurement of the impervious and nonimpervious land areas, the land class changes from 1993 to 2017 were increased by 35.20%, 75%, and 15% for the different cities in Kyrgyzstan.

**Figure 7:**
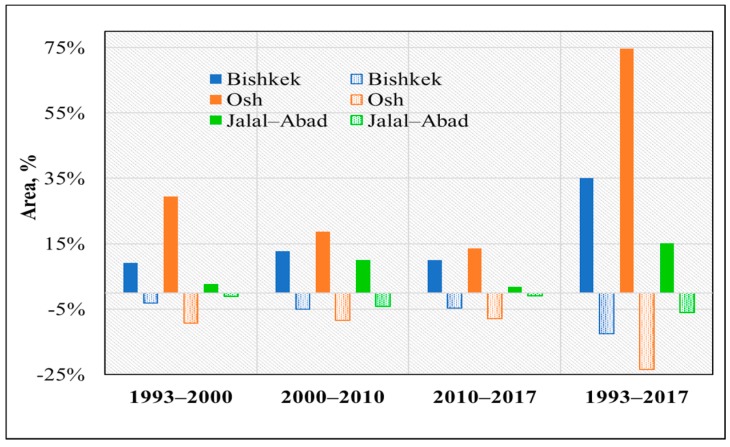
Changes in impervious and nonimpervious surfaces during 1993–2017.

Additionally, Figure 7 shows that the nonimpervious surface areas decreased significantly. Impervious surface areas increased by approximately 9.10%–35.20% for Bishkek, 29%–75% for Osh, and 3%–15% for Jalal-Abad during the study period. Nonimpervious surface areas decreased by 3%–12.49% for Bishkek, 8%–23% for Osh, and 1%–6% for Jalal-Abad.

### 4.3. Landscape Expansion Index (LEI)

**Figure 8:**
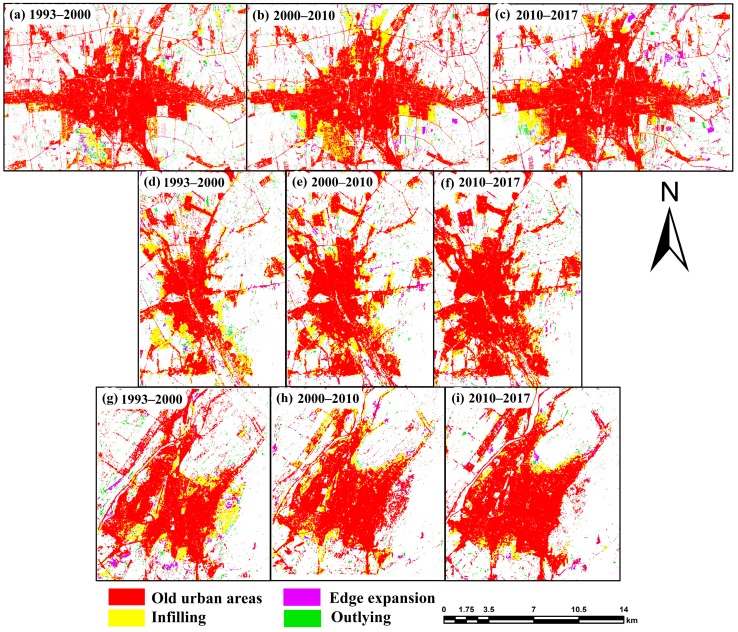
Spatial distribution of urban growth in the three main cities of Kyrgyzstan for the study period for (**a**–**c**) Bishkek, (**d**–**f**) Osh, and (**g**–**i**) Jalal-Abad for three different periods.

**Figure 9:**
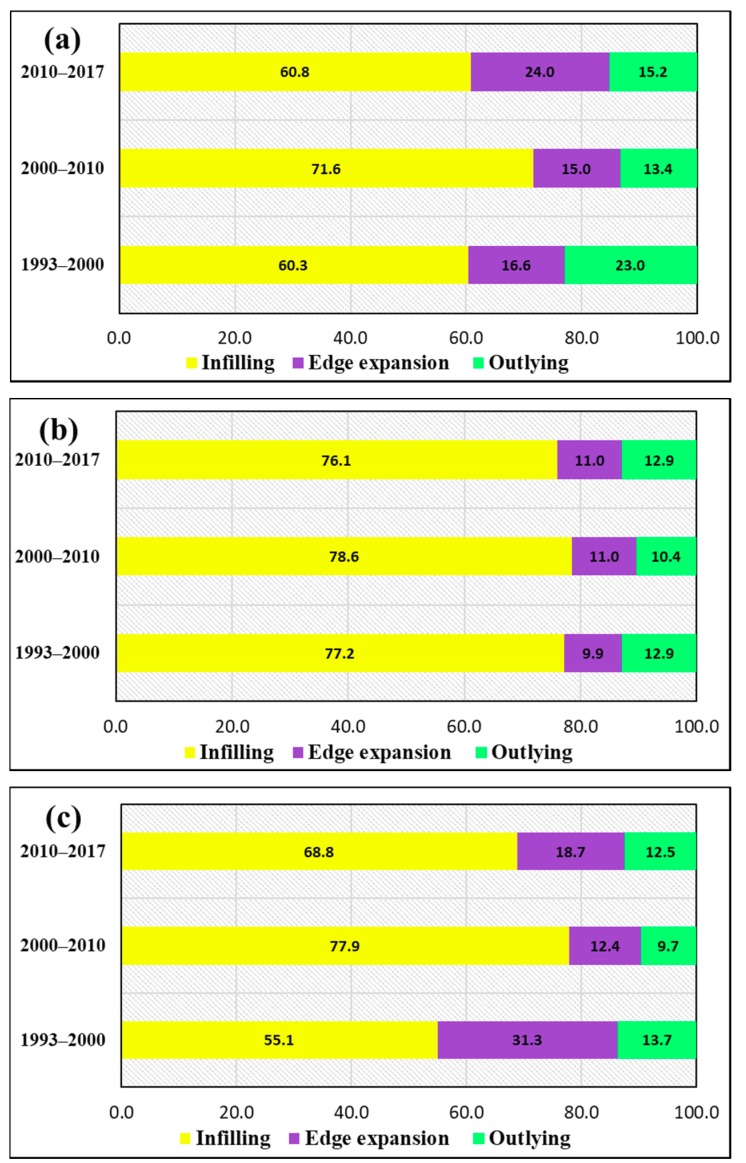
Percentages of area for the urban growth types in the three cities: (**a**) Bishkek, (**b**) Osh and (**c**) Jalal-Abad.

Detailed information about the urban types was obtained by using the LEI (Figure 8 and Figure 9). Figure 3 shows the contribution of the three urban patches in different periods. Throughout the 24-year period, urban growth was dominated by infilling. As illustrated in Figure 9a, from 1993–2000, infill growth accounted for a significant percentage at 60.3%, while edge expansion and outlying growth accounted for 16.6% and 23%, respectively.

From 2000 to 2010, edge expansion and outlying growth decreased due to 11.3% growth in the infilling patches. From 2010 to 2017, infilling growth decreased by 10.8%, followed by edge expansion by 8.9% and outlying growth by 1.9%. From Figure 9b, between 1993 and 2000, infilling growth accounted for nearly 77% of growth, during the 2000–2010 period, it increased by 1.4%, whereas in the last period, it decreased by 2.5%. Between 2000–2010 and 2010–2017, edge expansion and outlying growth changed from only 1.1% to 2.5%. In Figure 9c, for Jalal-Abad during the first period (1993–2000), infilling growth, edge expansion, and outlying growth accounted for 55%, 31.3%, and 13.7%, respectively; in the second period, infilling growth increased by 22.8%, whereas edge expansion and outlying growth decreased by 18.9% and 4%, respectively. In 2010–2017, edge expansion and outlying growth accounted for 9.1% less area than infilling growth.

### 4.4. Relationship between Impervious Surface Expansion and the Driving Factors

**Figure 10:**
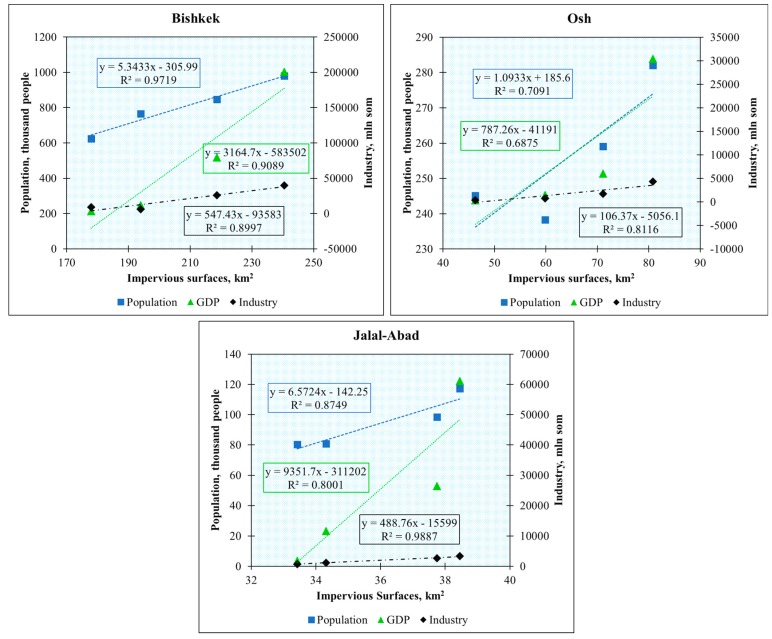
Correlation between impervious surface changes and population, gross domestic product (GDP), and industrial changes for the period 1993–2017.

Figure 10 shows a correlation in Bishkek during the period 1993–2017 with R^2^ = 0.97, which indicated the right balance between impervious surface area and population; the correlation between impervious surface area and GDP was R^2^ = 0.90; and the relationship between impervious surface area and industry was R^2^ = 0.89. For Osh, the correlation between impervious surface area and population was R^2^ = 0.70, between impervious surface area and GDP was R^2^ = 0.68, and between impervious surface area and industry was R^2^ = 0.81. Additionally, for Jalal-Abad, the correlations were R^2^ = 87, R^2^ = 0.80, and R^2^ = 0.98 between impervious surface area and population, GDP, and industry, respectively. Urban economic development is closely linked to urbanization. The environmental side of the problem is caused by the overpopulation of cities. Population and economic urbanization have positive (safety, employment, education, health, transport, social services) and negative (loss of natural habitat and ecosystem, land degradation, air pollution) impacts on cities and the environment at the same time [65].

## 5. Discussion

Urbanization processes in the regions of Kyrgyzstan were uneven. Considering the pattern of internal migration, a number of trends accompanying these processes should be highlighted: migration of the population from villages to cities; from regional cities to large cities (Bishkek, Osh, and Jalal-Abad); and from economically underdeveloped, disadvantaged regions to more developed, prosperous regions [66]. An increase in the number of people leads to the use of a large number of resources; the main burden is placed on the national power plants and heating and electric stations of Bishkek. Over the past 25 years, around the city of Bishkek, a ring of unplanned, chaotic residential areas has been built. Their number has reached 52. The city is “capturing” new territories around itself, mainly through the transformation of agricultural lands, as well as by reducing parks and green areas. In most residential areas, which have no infrastructure, social, or cultural facilities, single-story houses were built. The capital is the largest revenue-generating entity in the country. Of the revenue of the country’s total consolidated budget, the city of Bishkek accounts for more than 60%, which is due to the high concentration of the country’s manufacturing sector in the city. Bishkek produces approximately 20% of the country’s GDP [67].

The relationship between the mapped impervious surface areas and socio-economic changes in the three main cities and change statistics were also calculated for the GDP, industrial, and population data between 1993 and 2017.

Kyrgyzstan experienced different patterns of impervious surface expansion from 1993 to 2017. The impervious surface area increased quickly for the years 2000 to 2010 compared to 1990 to 2000. The spatial distribution of impervious area expansion in Bishkek, Osh, and Jalal-Abad was not similar. The built-up areas were mainly located in the center of the three main cities of the country. Bishkek had the largest area of unplanned impervious surface area increase during the study period from 1993 to 2017. The unplanned impervious surface areas increased from 26.2% (17,791.11 ha) to 35.4% (24,046.665 ha), 23.9% (4626.99 ha) to 41.8% (8079.66 ha), and 28.8% (3342.42 ha) to 33.1% (3844.62 ha) for Bishkek, Osh, and Jalal-Abad, respectively. Moreover, the properties of the unplanned impervious surfaces varied across cities and regions in Kyrgyzstan from 1993 to 2017, a phenomenon that was also described in many previous studies, despite some minor changes in the details [15]. This study mainly focused on impervious land changes in the three main cities of Kyrgyzstan; all cities expanded faster from 2000 to 2010 than from 1993 to 2000. From the above comparison and results, the spatio-temporal properties of impervious surface expansion found in this paper appear to be reliable. The study’s findings were different from other studies based on the scale of the period and the different definitions. Therefore, we examined the whole country and individual selected cities, including investigating the impacting factors of three cities in Kyrgyzstan. The results are important as they provide a new understanding of urbanization in Kyrgyzstan. The impervious surface expansion factors in Kyrgyzstan were defined by qualitative analysis. From this qualitative analysis, which indicated a positive relationship with impervious surface area expansion, some factors showed adverse effects. Based on the results, this finding indicates that initial industrial growth has hindered the construction of impervious surfaces. During the period ranging from 2000 to 2010, the construction industry in the study area focused on nonimpervious surface area expansion, which was subject to GDP growth.

The results showed that the impervious surface area increased significantly during the study period, which also had an impact on the country’s economy. The country’s economy, GDP, amount and actual use of foreign reserves, and capital income increased, mostly in the period from 1993 to 2017. Moreover, the growth rate of these economic factors topped the growth rate of impervious surfaces.

Spatial data and social-economic statistical data were used to quantitatively analyze different factors that affected urban land sprawl. Furthermore, statistical data showed that population, GDP, and industry factors significantly impacted urban expansion compared to other factors, such as topography, hydrology, and the effect of adjacent cities (particularly in cities near the capital) and land accessibility, which are significant factors but are difficult to measure [14]. Furthermore, spatial data are more reliable for local areas (i.e., small-scale cities), where statistical (socio-economic) data such as population, income, and investments are more suitable for small-scale studies than large-scale studies [26].

In this research, the influence of socio-economic factors was quantified, whereas the effects of the other factors on impervious surface expansion patterns were briefly discussed in previous studies [40,68,69]. The results for uncultivable population and GDP factors are the same as previous studies conducted in this area [14], while their impacts and parameters were defined and additionally itemized in this investigation. An earlier report demonstrated that impervious surfaces were related to urban arrangement and financial advancement because of the approach in Kyrgyzstan of “revitalizing the old industrial base”, characteristic interferences, and restrictions caused by the regional boundaries in the impervious surface expansion of three main cities in Kyrgyzstan from 1993 to 2017 [40]. To avoid gaps in the data on the fine-scale changes in impervious surfaces, high-spatial-resolution imaging software, such as QuikBird, GeoEye, and IKONOS, were used to extract different types of infrastructure and settlement development information, as well as hyperspectral data that had potential indicators for the extraction of spatial information from the ground. These data will be used as reference data for validation purposes in future work [70,71,72,73]. In this study, Sentinel-2 data with a spatial resolution of 10 m was used for validation and AA of land cover classifications extracted from Landsat images. Medium-resolution (30 m) land cover maps of Kyrgyzstan were drawn, and further analysis was conducted between impervious surface expansion and the declining farmland or nonurban land elements. This analysis is warranted to understand the qualities and impacts of urban land spread in the study area. In addition, coordinate causes, urban arrangements, and natural hindrances of urbanization should be explored [14]. Spatial variables (similar to separation for the downtown area), streams, farmland, and streets ought to be picked as components in the investigations, as they are more influential and expressive in space than spatial factors [74,75]. Cities in Kyrgyzstan underwent unique urban expansion from 1993 to 2017. GIS-integrated techniques were used to analyze land use type and landscape expansion [61]. An earlier study discovered that the infilling type was the dominant growth type for all cities. In Figure 8b,c, we can see that the southwest part of Bishkek developed more during 2000–2010 and 2010–2017 than during 1993–2000. In Osh, the infilling growth type increased in 2000–2010, but decreased in 2010–2017, followed by 2.5% outlying growth. In Figure 9c, for the period 2000–2010, edge expansion transitioned to infilling growth type (increasing to 22.8%, with a decrease of 9.1% from 2010–2017), followed by edge expansion and outlying growth.

Policy implications are outlined below:

1. The above results revealed that the impervious surface area increased from 1993 to 2017 in response to driving forces such as population, gross domestic product, and industry, implying that the government of Kyrgyzstan should have a clear policy and implement laws regarding integrated land use.
2. Urban planning policies should be based on “green growth”, which is defined in terms of economic growth and environmental sustainability, with a clear aim of creating a healthy environment that the public can enjoy without harming others. The economic growth of Kyrgyzstan is highly dependent on natural resources, especially on land, so sustainable use of these resources is quite important for the future generations. This suggests that the relationship between economic growth and urban expansion must be explored [12].
3. Bishkek, Osh, and Jalal-Abad are encompassing increasingly more land due to immense population pressure. Consequently, agricultural land, parks, and green zones are decreasing. Taking into account all these aspects, there is an urgent need for intensive land use approaches that account for more people, and to replace single-story houses with multistory infrastructure that covers the basic necessities of life (health, education, entertainment, etc.).
4. The results are subject to the fact that every year the population growth increases, especially in the city of Bishkek, since internal migration flows from village to city and natural processes of movement of labor resources play a dominant role. Increasing population also places pressure on the environment. The search for better employment is another factor in urban migration, as people move from remote areas to cities for a better income. For this, it is necessary to provide better living conditions along with better employment opportunities to the population living in remote areas. This will certainly help to minimize the migration pressure on the country’s major cities.

## 6. Conclusions

In this paper, the supervised maximum likelihood classification methodology was created to classify urban land cover types from RS satellite imagery. From the study results, it was found that the most attractive economies in Kyrgyzstan are Chui oblasts and the cities of Bishkek, Osh, and Jalal-Abad; the reasons were overcrowding and rapid urbanization in cities. Spontaneous intra-country population migration could become a serious threat to the sustainable economic and social development of the country. Due to the outflow of specialists from villages, the imbalance of the labour market, and the seizure of land and adjustment on the periphery of cities, an increase in the load on the social infrastructure of cities has appeared. Due to the population migration and lack of proper land use planning, impervious surfaces have increased randomly in different cities in Kyrgyzstan. An analysis in this study showed that the impervious surface areas increased by from 9.10% to 35% in Bishkek, from 29.4% to 75% in Osh, and from 3% to 15% in Jalal-Abad from 1993 to 2017, which came from natural vegetation and bare land. The urban population, gross domestic product (GDP), and industry factors were strongly positively associated with the impervious surface area growth. A new sector of economic activity has appeared in the country, the revenue of which is amounts to approximately a quarter of the GDP. The evaluation of demographics of Bishkek, Osh, and Jalal-Abad showed that the population increased by approximately 56% from 1993 to 2017. The most productive lands are being converted into concrete lands. These trends have not yet been stopped and could undoubtedly increase in the future unless an appropriate land use plan is defined. The proper land uses for urban development should be defined by considering the population’s different interests and needs. We employed the LEI to characterize urban expansion in Bishkek, Osh, and Jalal-Abad during three periods (1993–2000, 2000–2010, and 2010–2017). The urban growth dynamics were described by the relative dominance of infilling, edge expansion, and outlying growth modes across the landscape, in which infill and edge expansion were the dominant growth types. Infilling growth was initially managed by residential land centered on the original cities. There is a need for more focus on increasing or protecting the natural vegetation and green land areas. The results also showed significant a decrease in agriculture. It is recommended that urban expansion should be focused on bare land or desert areas if these exists instead of agricultural land, which provides food security for the population of the country. The results of this study will be valuable to local governments when developing sustainable land use and urban development plans.

## Figures and Tables

**Table 1:** List of Landsat data with their numbers of bands. Note: TM = Thematic Mapper; ETM + = Enhanced Thematic Mapper Plus; OLI = Operational Land Imager; USGS = United States Geological Survey.

Satellite	Sensor	Imagery Date	Spatial Resolution (Meters)	Path/Row	Cloud Cover (Percent)	Band	Source
Landsat 5	TM	6/10/1993	30	151/30	6	7	USGS glovis.usgs.gov
		6/10/1993		151/32	1		
Landsat 7	ETM+	8/24/2000	30	151/30	6	6, 7	
				151/32	0		
Landsat 5	TM	8/12/2010	30	151/30	4	7	
		8/28/2010		151/32	1		
Landsat 8	OLI	8/12/2017	30	151/30	2.4	8	
		7/11/2017		151/32	2.93		

**Table 2:** Overall accuracy (OA) and kappa coefficients (KCs) for the classifications of Landsat TM, ETM, and 8 OLI data from 1993 to 2017, and for Sentinel-2 for 2017.

Class	Landsat Cities	1993		2000		2010		2017		Sentinel-2 (2017)	
		PA	UA	PA	UA	PA	UA	PA	UA	PA	UA
IS	Bishkek	93.3	93.3	93.3	96.6	93.3	90.3	93.3	87.5	100	93.8
	Osh	73.3	88	100	78.9	100	88.2	96.7	85.5	100	68.2
	Jalal-Abad	93.3	96.6	76.7	79.3	90	93.1	93.3	93.3	96.7	78.4
NV	Bishkek	96.7	85.3	96.7	59.2	93.3	77.8	93.3	65.1	90	90
	Osh	96.7	64.4	90	75	100	100	100	65.1	93.3	100
	Jalal-Abad	96.7	80.6	90	61.4	96.7	53.7	100	83.3	100	93.8
BL	Bishkek	90	100	40	100	83.3	100	60	100	90	93.1
	Osh	93.3	96.6	90	100	100	100	50	100	93.3	100
	Jalal-Abad	86.7	96.3	66.7	95.2	20	100	80	100	73.3	100
WB	Bishkek	96.7	100	100	100	93.3	100	90	100	96.7	100
	Osh	70	100	63.3	100	86.7	100	83.3	100	66.7	100
	Jalal-Abad	90	96.4	86.7	100	100	96.8	100	100	96.7	100
Overall	Bishkek	94.2		82.5		90.8		84.2		94	
	Osh	83.3		85.8		96.7		82.5		88	
	Jalal-Abad	91.7		80		76.7		93.3		92	
Kappa	Bishkek	92		77		87.8		78.9		92	
	Osh	78		81		96		77		84	
	Jalal-Abad	89		73		69		91		89	
